# Axillo-caval extra-anatomic venous bypass creation via direct percutaneous puncture of the superior vena cava

**DOI:** 10.1186/s42155-025-00518-1

**Published:** 2025-02-08

**Authors:** Stephan S Leung, Patrick Lee, Anthony N Hage, Robert W Ford

**Affiliations:** Division of Interventional Radiology, Department of Radiology, Thomas Jefferson University Hospital, Philadelphia, PA USA

**Keywords:** Hemodialysis, Venous occlusion, Stenting, Extra-anatomic bypass, Swelling

## Background

Patients with chronic catheters for hemodialysis access have a 10% risk for developing central venous obstruction [1]. This risk increases for catheters placed on the left side [1]. Although patients with central venous obstruction may be asymptomatic, about 50% of patients with significant obstruction will develop ipsilateral upper extremity swelling, tenderness, and/or pain [1]. In symptomatic patients, venous recanalization can restore anatomic central venous flow with improvement in symptoms and overall quality of life. Central venous occlusions may be treated with either endovascular or surgical techniques [2, 3]. However, long segment central venous occlusions are often difficult to treat due to the difficulty in recanalizing the entire length of the occlusion via endovascular means. Percutaneous extra-anatomic venous bypass creation as a technique has been described in patients with symptomatic upper extremity edema secondary to chronic deep venous occlusion [4]. However, when the venous occlusion extends to the superior vena cava (SVC), extra-anatomic bypass is challenging due to work within the mediastinum and near critical vascular structures. This case highlights the successful creation of an extra-anatomic venous bypass via direct percutaneous puncture of the SVC for symptomatic relief of severe left upper extremity (LUE) swelling and pain.

## Case Report

A 32-year-old male with a history of end stage renal disease on hemodialysis presented with worsening isolated LUE swelling and pain. He has a long-standing history of challenging venous access, including tunneled dialysis catheters in the femoral veins and on presentation was receiving dialysis via right chest wall tunneled dialysis catheter placed through an occluded brachiocephalic vein (BCV) stent. Work-up imaging demonstrated long segment left axillary vein (AV) through BCV occlusion. An endovascular attempt at left AV through BCV recanalization was unsuccessful due to inability to cross the long-segment occlusion. Vascular Surgery was consulted for placement of a left AV to SVC bypass, but patient declined surgery and elected to manage his symptoms conservatively.

Over the course of a year, the patient had multiple hospital admissions due to chronic co-morbidities which included frequent clotting of the dialysis catheter, several episodes of bacteremia and ongoing abdominal pain. During one hospitalization in March 2023, the patient experienced an acute worsening of left upper extremity swelling and new onset severe left arm pain. Ultrasound imaging ruled out acute deep venous thrombosis and showed findings of stable chronic left central venous occlusion. Due to worsening isolated left upper extremity swelling, a repeat recanalization attempt was undertaken.

**Fig. 1:**
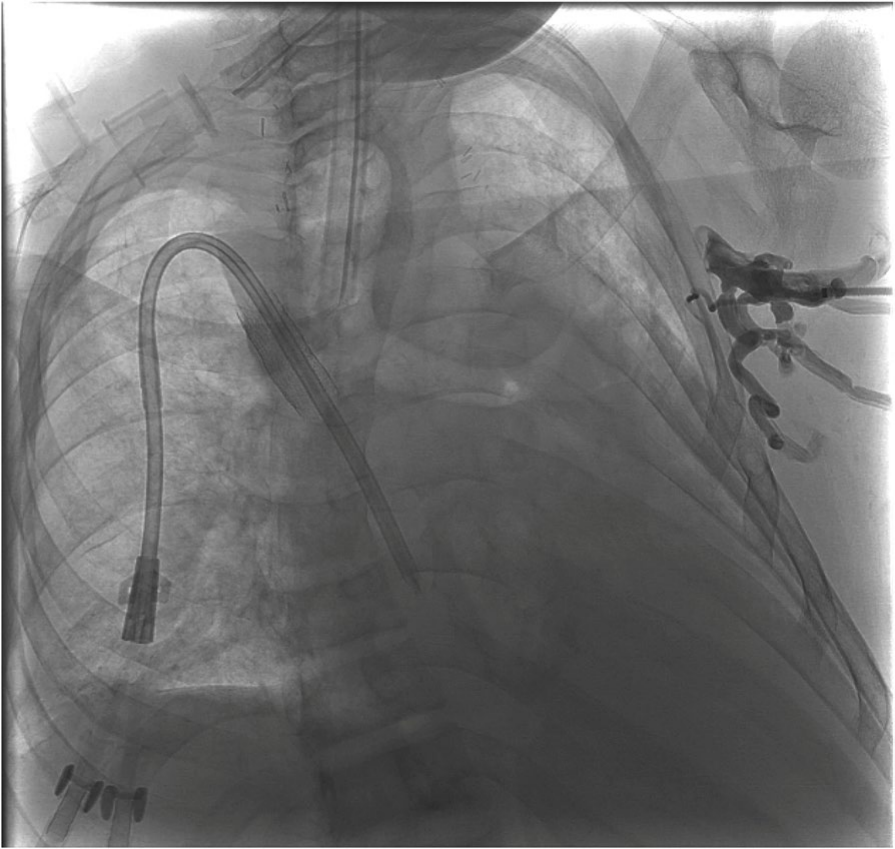
Digital subtraction angiography with a sheath from the left upper extremity demonstrates occlusion of the left subclavian and brachiocephalic veins with prominent left chest wall collateral veins

The patient was placed under general anesthesia. A 6 French sheath was placed into the left brachial vein (BV) and a 10 French sheath was placed in the right common femoral vein (CFV). Several unsuccessful attempts were made to cross the occluded left AV through BCV using glide wires and catheters (Fig. 1). It became clear that sharp recanalization techniques would be necessary. We considered supraclavicular percutaneous extra-anatomic recanalization (SPEAR), which has been previously described [5]; however, access from the patent peripheral vein to the patent central vein was not feasible due to the long-segment occlusion. While SPEAR was not possible, we had appropriate ultrasound visualization for direct access to the SVC. This prompted us to consider a percutaneous extra-anatomic venous bypass.

**Fig. 2:**
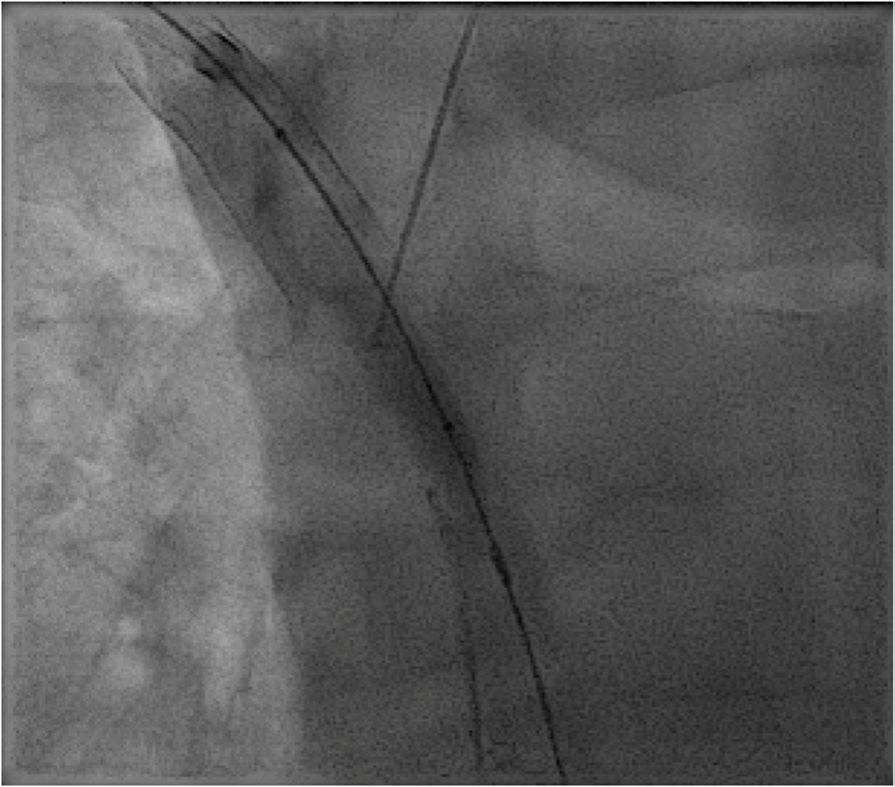
Magnified fluoroscopic image of the chest demonstrates a balloon catheter inflated in the SVC central to a right BCV stent which was used as a target. There is a needle directed from a left supraclavicular approach for direct percutaneous transmediastinal puncture into the SVC

**Fig. 3:**
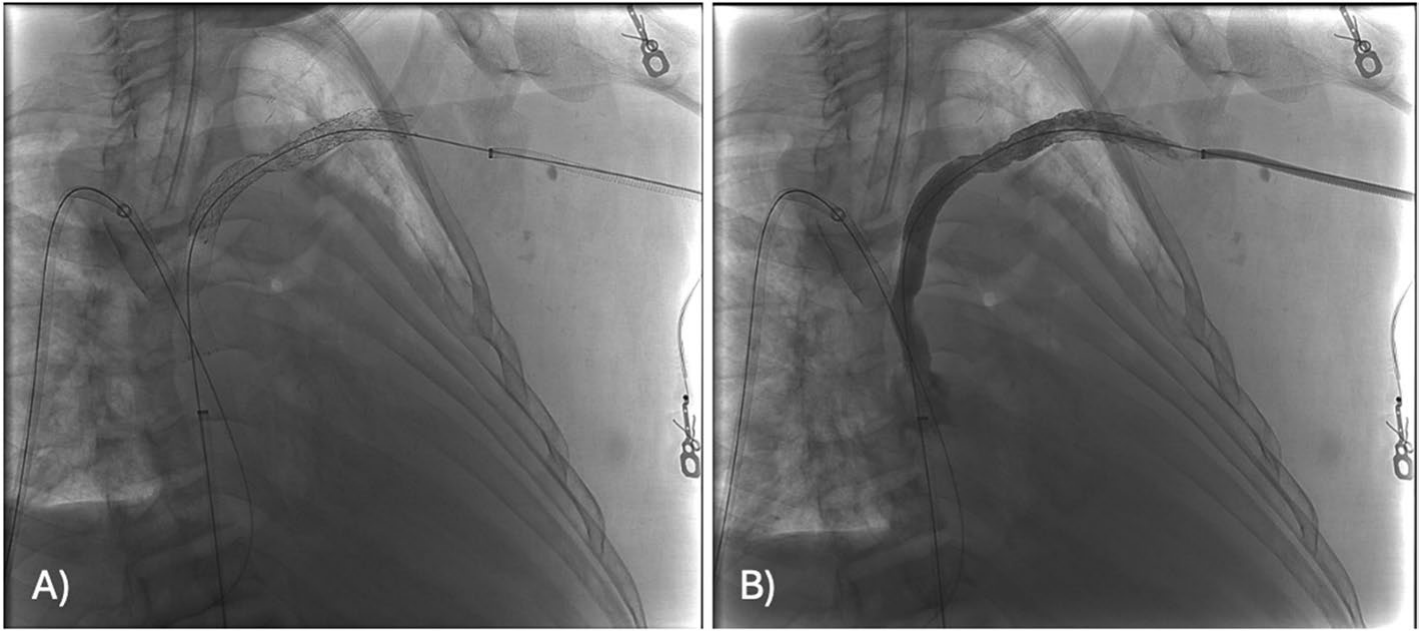
Fluoroscopic images of the chest after deployment of the left subclavian through SVC stent before (**A**) and after (**B**) contrast injection demonstrate stent patency with brisk flow and without filling defect

The existing right chest wall tunneled dialysis catheter was exchanged for a 12 French sheath. An 8 mm Conquest balloon was placed into the upper SVC as a target through this sheath access. Under initial ultrasound guidance, a 20-gauge INRAD needle was advanced from a left supraclavicular percutaneous approach to the central margin of the right BCV stent. Under fluoroscopic guidance the needle was used to puncture the target balloon in the SVC (Fig. 2). A mandril wire was advanced into the inferior vena cava (IVC) and the needle was exchanged for a 5 French GrebSet (Teleflex). An over-the-wire tract-o-gram was performed through the introducer set to ensure no critical vascular structures were transgressed. Using a 21-gauge needle, the central left AV was percutaneously accessed under ultrasound guidance from a more lateral position in the supraclavicular fossa. A 5 French sheath was placed and a GooseNeck snare (Medtronic, Inc) was used to externalize a glide wire originating from the left BV access. A subcutaneous tunnel was created between the percutaneous AV access to the percutaneous SVC access site in the supraclavicular fossa using a blunt tip needle and a 6 French peel away sheath. Another 6 French peel away sheath was advanced over the mediastinal access and into the SVC. The glide wire originating from the brachial vein access was then advanced through the subcutaneous tunnel, peel-away sheath, and into the IVC. The glide wire was then snared in the IVC from the femoral sheath to establish through-and-through access from the left upper extremity to the right groin. The glide wire was exchanged for a super stiff Amplatz wire. Intravascular ultrasound (IVUS) was used to characterize the length of occlusion and determine appropriate stent sizing/length. A 10 mm x 15 cm Viabahn covered stent graft (Gore Medical) was deployed from the SVC to the left AV. The stent graft was dilated to 10 mm using Mustang balloons. Repeat venography and IVUS were performed showing chronic post thrombotic change peripheral to the landing zone of the Viabahn stent (Fig. 3). Therefore, a 10 mm Abre stent (Medtronic, Inc) was placed to overlap with the Viabahn stent and extend into the central left AV. The right tunneled dialysis catheter was replaced.

The patient was transferred to the medical intensive care unit (MICU) where he was extubated and had an unremarkable postoperative course. He was placed on Eliquis and was scheduled for clinic follow-up in 1 month post procedure with planned surveillance outpatient ultrasound imaging to monitor venous bypass patency. He was discharged from the hospital on post-procedure day 4 in stable condition.

**Fig. 4:**
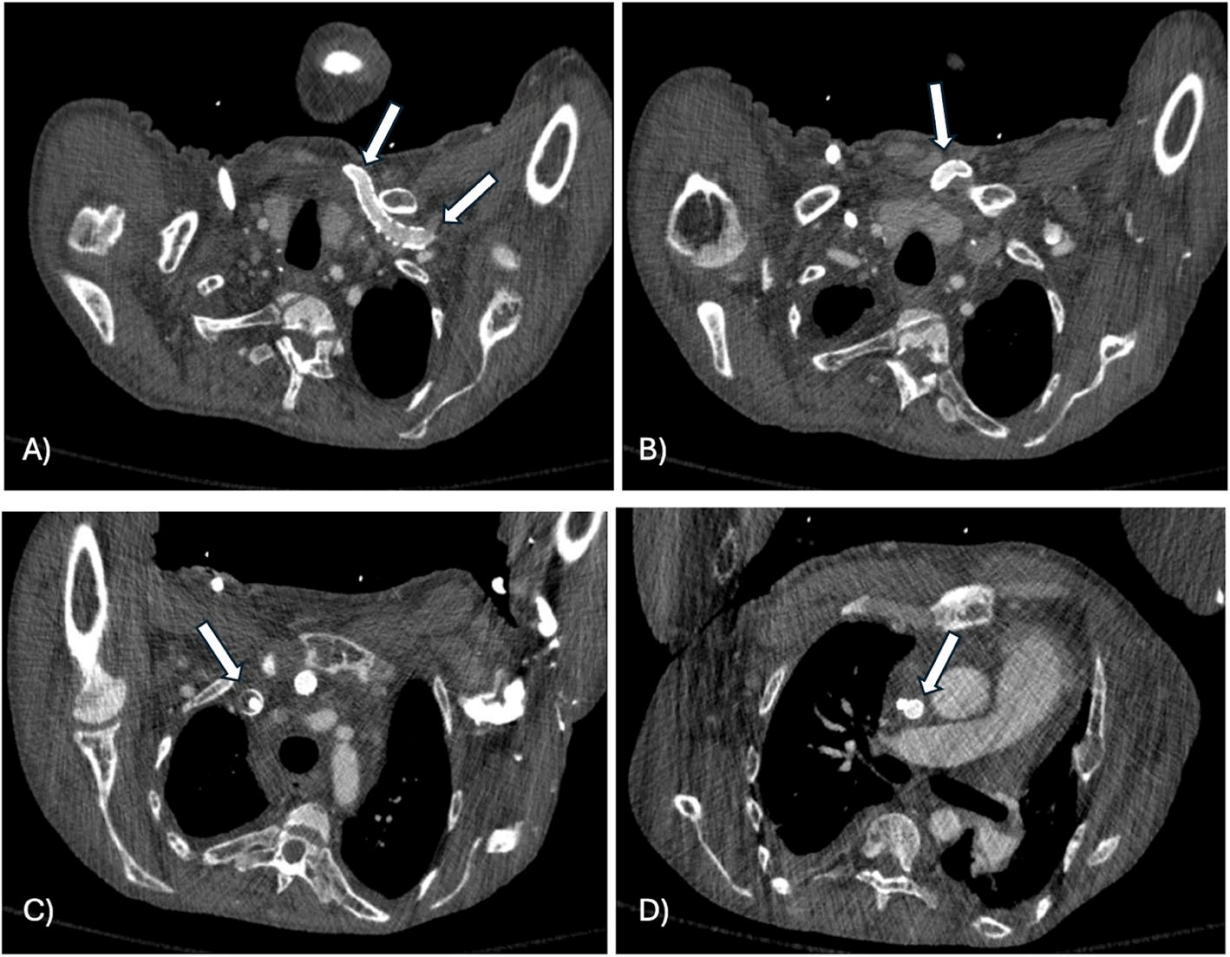
Serial axial contrast enhanced CT images of the chest from cranial (**A**) through caudal (**D**) show patency of the left subclavian through SVC stents (white arrows) at 3 months without significant filling defect

The patient missed his outpatient imaging ultrasound and follow-up clinic visit. He was admitted to the hospital 3 months later for abdominal pain. At that time, he had resolution of his left upper extremity swelling and pain. Computed tomography (CT) imaging of the chest (performed to rule out pulmonary embolism) demonstrated patency of the left upper extremity extra-anatomic bypass graft (Fig. 4). He had several additional admissions to the hospital and passed away a year later due to complications from his other medical co-morbidities.

## Discussion

Central venous occlusion is a challenging and serious problem in hemodialysis patients. For symptomatic patients, endovascular therapy is the first line treatment for central venous occlusion as surgery can be associated with significant morbidity [1].

Endovascular management involves recanalization of the diseased segment of vein coupled with angioplasty and/or stenting. Angioplasty has a variable success rate; a study by Kovalik et al. found a technical failure rate of 7%, with more than 50% improvement in 70% of patients with central venous obstruction and less than 50% improvement in 23% of patients [6]. This was due to two types of described venous lesions: nonelastic lesions that respond well to angioplasty and elastic lesions that did not respond to angioplasty. Primary patency after angioplasty also demonstrates a range of variability. A study by Bakken et al. which looked at 47 patients demonstrated a 3-month patency of 58%, 6-month patency of 45%, and 12-month patency of 29% after angioplasty [7].

Stenting is used to assist in establishing and maintaining venous patency after opening a stenosis or occlusion. Primary patency rates after stenting are also quite variable. In the same study by Bakken et al., they found patients who underwent bare metal stenting had a 3-month patency of 65%, 6-month patency of 54%, and 12-month patency of 45% [7]. A more recent study by Gür et al. in 2019 compared primary patency rates between angioplasty alone versus stent placement [8]. The technical success rate was 94%. In 141 patients, the primary patency rate of stent placement was higher than that of angioplasty alone at 12-months (58.7% versus 42.4%), 24-months (41.9% versus 36.6%), and 60-months (27.9% versus 20.2%).

Failure of endovascular recanalization is due to the impossibility of crossing a vascular occlusion. In these patients, an extra-anatomic bypass can be created to allow for restoration of central venous flow. This can be performed through several methods: via direct cannulation of the patent veins adjacent to the diseased segment, via existing central venous access, or via an “inside-out” approach where external access is obtained via a needle from the SVC to the right supraclavicular fossa [4, 9]. The difficulty of these techniques, particularly for left-sided occlusions, include safely navigating around critical vascular structures in the mediastinum. A newly described technique, called SPEAR, uses ultrasound guidance to visualize the patent venous lumens peripheral and central to the occlusion, as well as visualization of arterial structures [5]. A needle is then passed through the peripheral lumen and then, using a combination of sonographic and fluoroscopic guidance, is advanced in the central lumen thereby creating a bypass. However, the challenge in using the SPEAR technique in this case was the anatomic impossibility of passing a needle from the left AV to the SVC. Therefore, several combined techniques were used in the creation of the described extra-anatomic bypass.

## Conclusion

**Fig. 5:**
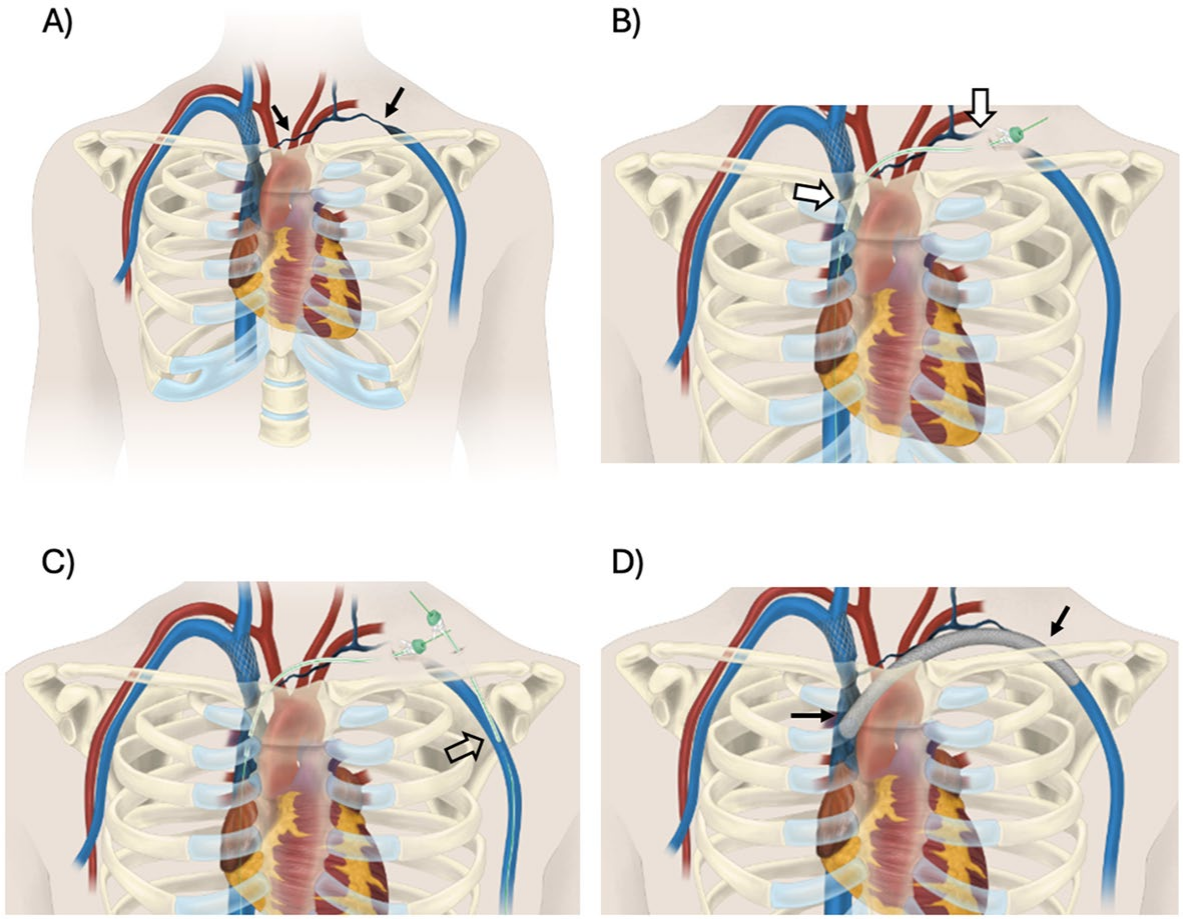
Pictorial representation of the percutaneous extra-anatomic venous bypass procedure. **A** There is long segment occlusion of the left subclavian and brachiocephalic vein (black arrows) leading to symptomatic left upper extremity swelling. **B** Trans-mediastinal sheath access (white arrows) is obtained in an antegrade fashion into the SVC via a percutaneous supraclavicular approach. **C** Retrograde sheath access is obtained into the left axillary vein (open arrow). **D** After tunneling underneath the skin and obtaining through and through access from the SVC through the axillary vein, a covered stent was deployed (black arrows) allowing for central venous drainage via the new extra-anatomic bypass

This case highlights a heroic attempt at the creation of an extra-anatomic venous bypass via direct puncture of the SVC (Fig. 5) to bypass a long segment LUE venous occlusion which was previously unable to be recanalized through an endovascular approach. Due to the inherent challenges, this procedure should be reserved for extreme cases in management. This case proves the feasibility of performing such a technique for patients with complex central venous occlusion using sonographic and fluoroscopic guidance to avoid critical vascular structures in the mediastinum.

## Electronic supplementary material

Supplementary Material 1.

## Abbreviations

SVC: Superior vena cava
LUE: Left upper extremity
BCV: Brachiocephalic vein
AV: Axillary vein
BV: Brachial vein
CFV: Common femoral vein
SPEAR: Supraclavicular percutaneous extra-anatomic recanalization
IVC: Inferior vena cava
IVUS: Intravascular ultrasound
MICU: Medical intensive care unit
CT: Computed tomography
