# Supplementary material for: Axillo-caval extra-anatomic venous bypass creation via direct percutaneous puncture of the superior vena cava
Source: CVIR Endovasc. 2025 Feb 8;8:12. doi: 10.1186/s42155-025-00518-1 (PMC11807030; doi:10.1186/s42155-025-00518-1)
Supplement: Supplementary file 1 — Supplementary Material 1. [file 42155_2025_518_MOESM1_ESM.docx]

**Declarations:**

**Ethics Approval and Consent to Participate:** Institutional review board approval was waived.

**Consent for Publication:** This study was reviewed by the Jefferson Privacy Office and the need for informed consent was waived.

**Availability of Data and Material:** Not applicable.

**Competing Interests:** The authors declare that they have no competing interests.

**Funding:** This study was not supported by any funding.

**Authors’ Contributions:** All authors contributed equally to the preparation of this study and manuscript.

**Acknowledgements:** Not applicable.
